# Motor Skill Learning-Induced Functional Plasticity in the Primary Somatosensory Cortex: A Comparison Between Young and Older Adults

**DOI:** 10.3389/fnagi.2020.596438

**Published:** 2020-11-25

**Authors:** Claudia Predel, Elisabeth Kaminski, Maike Hoff, Daniel Carius, Arno Villringer, Patrick Ragert

**Affiliations:** ^1^Max Planck Institute for Human Cognitive and Brain Sciences, Leipzig, Germany; ^2^Institute for General Kinesiology and Exercise Science, University of Leipzig, Leipzig, Germany; ^3^Berlin School of Mind and Brain, Mind Brain Body Institute, Humboldt University of Berlin, Berlin, Germany

**Keywords:** aging, motor learning, sensorimotor integration, somatosensory evoked potential (SEP), functional plasticity

## Abstract

While in young adults (YAs) the underlying neural mechanisms of motor learning are well-studied, studies on the involvement of the somatosensory system during motor skill learning in older adults (OAs) remain sparse. Therefore, the aim of the present study was to investigate motor learning-induced neuroplasticity in the primary somatosensory cortex (S1) in YAs and OAs. Somatosensory evoked potentials (SEPs) were used to quantify somatosensory activation prior and immediately after motor skill learning in 20 right-handed healthy YAs (age range: 19–35 years) and OAs (age range: 57–76 years). Participants underwent a single session of a 30-min co-contraction task of the abductor pollicis brevis (APB) and deltoid muscle. To assess the effect of motor learning, muscle onset asynchrony (MOA) between the onsets of the contractions of both muscles was measured using electromyography monitoring. In both groups, MOA shortened significantly during motor learning, with YAs showing bigger reductions. No changes were found in SEP amplitudes after motor learning in both groups. However, a correlation analysis revealed an association between baseline SEP amplitudes of the N20/P25 and N30 SEP component and the motor learning slope in YAs such that higher amplitudes are related to higher learning. Hence, the present findings suggest that SEP amplitudes might serve as a predictor of individual motor learning success, at least in YAs. Additionally, our results suggest that OAs are still capable of learning complex motor tasks, showing the importance of motor training in higher age to remain an active part of our society as a prevention for care dependency.

## Introduction

We are confronted with an aging society worldwide (He et al., 2016), which in turn has a huge impact on the health care and social system. Hence, great effort over the last decades has been put into understanding how to improve healthy and successful aging. However, even during healthy aging, some basic functions undergo age-related decline. Examples of the age-related decline in motor function are prolonged reaction times (Morgan et al., 1994; Salthouse, 1996; Cuypers et al., 2013; Hoff et al., 2015), diminished inter-limb coordination (Serrien et al., 2000; Fujiyama et al., 2009; Van Impe et al., 2009; Goble et al., 2010; Solesio-Jofre et al., 2014), decline in balance performance (Iosa et al., 2014; Kaminski et al., 2017), and reduced precision in movement execution (Stewart et al., 2014). Considering somatosensory function, previous studies have shown poorer performance of older adults (OAs) in tactile acuity like two-point discrimination (Franco et al., 2015) and haptic perception (Norman et al., 2016), as well as diminished proprioceptive skills (Herter et al., 2014). Interestingly, age-related alterations in motor and somatosensory regions are often related. For example, stronger brain activation and recruitment of additional areas during motor tasks in OAs compared with younger adults (YAs) (Ward and Frackowiak, 2003; Heuninckx et al., 2008; Berchicci et al., 2012) are not only limited to the motor region but also involve somatosensory areas (Heuninckx et al., 2008). On a functional level, impaired somatosensory skills are associated with higher risks of falls (Lord et al., 1999). This functional relation between both areas can be explained by the strong interconnectedness of motor and somatosensory systems *via* neuronal cortico-cortical projections (Porter, 1992, 1997), which are potentially mediated by ⋎-amino butyric acid (GABA) transmission (Pleger et al., 2003).

Plastic changes in the primary somatosensory cortex (S1) can be investigated non-invasively using somatosensory evoked potentials (SEPs). Deriving SEPs under varying conditions makes it possible to investigate immediate changes following sensory processes (Angel et al., 1984), and combining SEP measurements with motor behavior provides a technique to quantify neural activation in S1 following motor learning. Several studies have shown that repetitive synchronized movements induce plastic changes not only in the primary motor cortex (M1) (Cohen et al., 1995; Liepert et al., 1999; Tegenthoff et al., 1999, 2004) but also in S1 (Schwenkreis et al., 2001; Pleger et al., 2003). These findings correspond to a variety of human brain mapping studies in YAs describing activation of S1 after motor task execution (Halsey et al., 1979; Kawashima et al., 1993; Kim et al., 1993; Rao et al., 1993; Mattay et al., 1998) or motor learning (Schwenkreis et al., 2001; Andrew et al., 2015). More specifically, mainly N20 and N20-P25 amplitudes of the SEP signal, representing components generated in S1, are shown to be enhanced after learning a repetitive motor task (Andrew et al., 2015). In OAs, it is known that SEP amplitudes increase as a function of aging (Pellicciari et al., 2009). Furthermore, the same study also found that N20–P25 amplitudes increase after a plasticity-enhancing intervention in OAs but not in YAs, suggesting that specific neuronal circuits are more prone to plasticity induction in older age (Pellicciari et al., 2009).

Even though it is known that the somatosensory system is highly connected and that loss of function in M1 and S1 in older age is not independent, it is not known if learning a novel motor task in older age also leads to functional adaptations of the somatosensory system. Therefore, the primary aim of this study was to investigate the functional changes in S1 processing in YAs and OAs before and after motor learning. A repetitive motor co-contraction task of thumb and arm muscles was used to establish a model of motor learning (Pleger et al., 2003), with the time difference between muscle activities serving as a marker for motor learning. SEP measurements before and after the motor task were used to quantify functional changes in S1 processing. Based on previous findings, we hypothesized that (1) motor learning is associated with SEP amplitude enhancement mainly in the N20 and N20/P25 component. Furthermore, we hypothesized that OAs in comparison to YAs show: (1) less synchronized muscle activity after a 30-min learning period, indicating smaller learning capacities (Stewart et al., 2014), and (2) higher baseline SEPs, with bigger changes in SEP amplitudes after motor training (Pellicciari et al., 2009). Furthermore, we wanted to investigate whether specific components of baseline SEP amplitudes might be used as a predictor for the amount of motor learning (Solesio-Jofre et al., 2018).

## Materials and Methods

### Subjects

A total number of 20 OAs (age range: 57–76 years) and 20 YAs (age range: 19–35 years) participated in the present study. All participants were right-handed, as assessed by laterality quotient (LQ) with the Edinburgh Handedness Scale (Oldfield, 1971). To exclude the presence of any neurological disease, all participants underwent a detailed neurological examination prior to the testing phase. This examination included a short review of the individual medical history (anamnesis); assessment of muscle strength and tone, gait, and posture; and evaluation of meningism signs. The cranial nerves were assessed, as well as the proprioceptive muscle reflexes including biceps, triceps, brachioradialis, patellar, and Achilles tendon reflexes. To exclude pathological pyramidal signs, Babinski reflex was performed. Furthermore, we assessed the function of the sensory system by provoking sensations of fine touch and pain. Testing for dysmetria, dysdiadochokinesis, ataxia, and intention tremor assessed the cerebellar function. Lastly, an orientating examination of the heart, lungs, and abdomen was carried out including auscultation, palpation, as well as measuring of blood pressure and heart rate. None of the participants showed any signs of neurological disease, and all of them were free of neurological or psychiatric medication. Before and after the experiment, participants rated their level of attention (1 = not attentive, 10 = very attentive), fatigue (1 = very tired, 10 = not tired at all), and discomfort (1 = no discomfort, 10 = strong discomfort) on a visual analog scale (VAS). Additionally, all participants were asked about their physical activity levels, prior experiences with musical instruments, and quality of sleep prior to the testing using a standardized questionnaire. Participants currently or formerly playing instruments on a professional level or with extensive sports participation were not included in the study, since musical or sports expertise has been shown to alter the sensorimotor system (Gaser and Schlaug, 2003; Hosoda and Furuya, 2016; Raichlen et al., 2016). A Mini Mental State Examination (MMSE; Folstein et al., 1975) performed with the OAs to test for dementia showed no signs of cognitive impairment (see Table 1 for further details). All participants gave a written informed consent before participation. The study was approved by the local ethics committee of the University of Leipzig and performed according to the Declaration of Helsinki.

**Table 1:** Group characteristics.

**Group**	**Age (years)**	**Gender (f/m)**	**LQ**	**Sports/week (h)**	**Sleep (h)**	**MMSE**
YA (*n* = 20)	25.70 ± 0.76	11/9	78.00 ± 3.42^*^	3.85 ± 0.69^*^	7.63 ± 0.22	-
OA (*n* = 20)	67.05 ± 1.21	10/10	94.95 ± 0.26^*^	1.75 ± 0.40^*^	7.55 ± 0.20	28.95 ± 0.26

*Gender (f/m): female/male number of participants. LQ: laterality quotient, score range −100 = full left-handed to +100 = full right-handed. MMSE: Mini Mental State Examination, total score range of 0–30; cutoff score for exclusion: ≤ 26*.

* *indicates significant differences (p < 0.05) in this variable between groups. All values are depicted as mean ± standard deviation of the mean. OA, older adults, YA, young adults*.

### Experimental Procedure

#### Co-contraction Task and Training

The aim of the present study was to learn a novel movement consisting of a co-contraction of the right abductor pollicis brevis (APB) and right deltoid muscle. This task was used in previous studies [e.g., Schwenkreis et al., 2001; Tegenthoff et al., 2004; Choi et al., 2016; see also Pleger et al. (2003), for illustration of the motor task] and shows robust learning effects over the time course of a single training session. During task performance, subjects were seated on a chair in a relaxed position with the monitor of the electromyography (EMG) equipment in front (Nihon Kohden Neuropack X1, Rosbach, Germany). The right upper arm was held in a relaxed position close to the right flank of the body, while the forearm and the hand were placed in front of the body in a right-angled position toward the upper arm. The aim was to perform an abduction of the right thumb and an elevation of the right upper arm as simultaneously as possible with APB moving first. The subjects were instructed to act on an acoustic signal and to make brisk and short movements of both muscles. Over 30 min, three co-contractions per minute had to be performed, resulting in a total of 90 movements in 30 min. To investigate motor learning, we measured the time differences between the onsets of the contractions of both muscles, termed “muscle onset asynchrony” (MOA), during each single co-contraction (Pleger et al., 2003) using EMG monitoring from surface electrodes. MOA served as the primary outcome measure for the individual motor learning success. For EMG measurements, two electrodes were attached to the muscle belly of the APB and deltoid muscle. Electrodes were positioned according to standard belly–tendon montage for bipolar recordings. For EMG recordings of the APB, the active electrode was positioned on the middle of the muscle belly of the APB. The electrode for the deltoid muscle was placed over the mid-acromial part of the muscle. An electrode at the right forearm served as ground electrode. EMGs were acquired with a band-pass filter between 10 and 5,000 Hz digitized with a sampling rate of 10,000 Hz (sampling interval 100 μs). EMGs were recorded non-continuously and stored for offline analysis. The subject was informed about the time difference after each movement in order to establish an auditory feedback.

#### Somatosensory Evoked Potential Measurements

**Figure 1:**
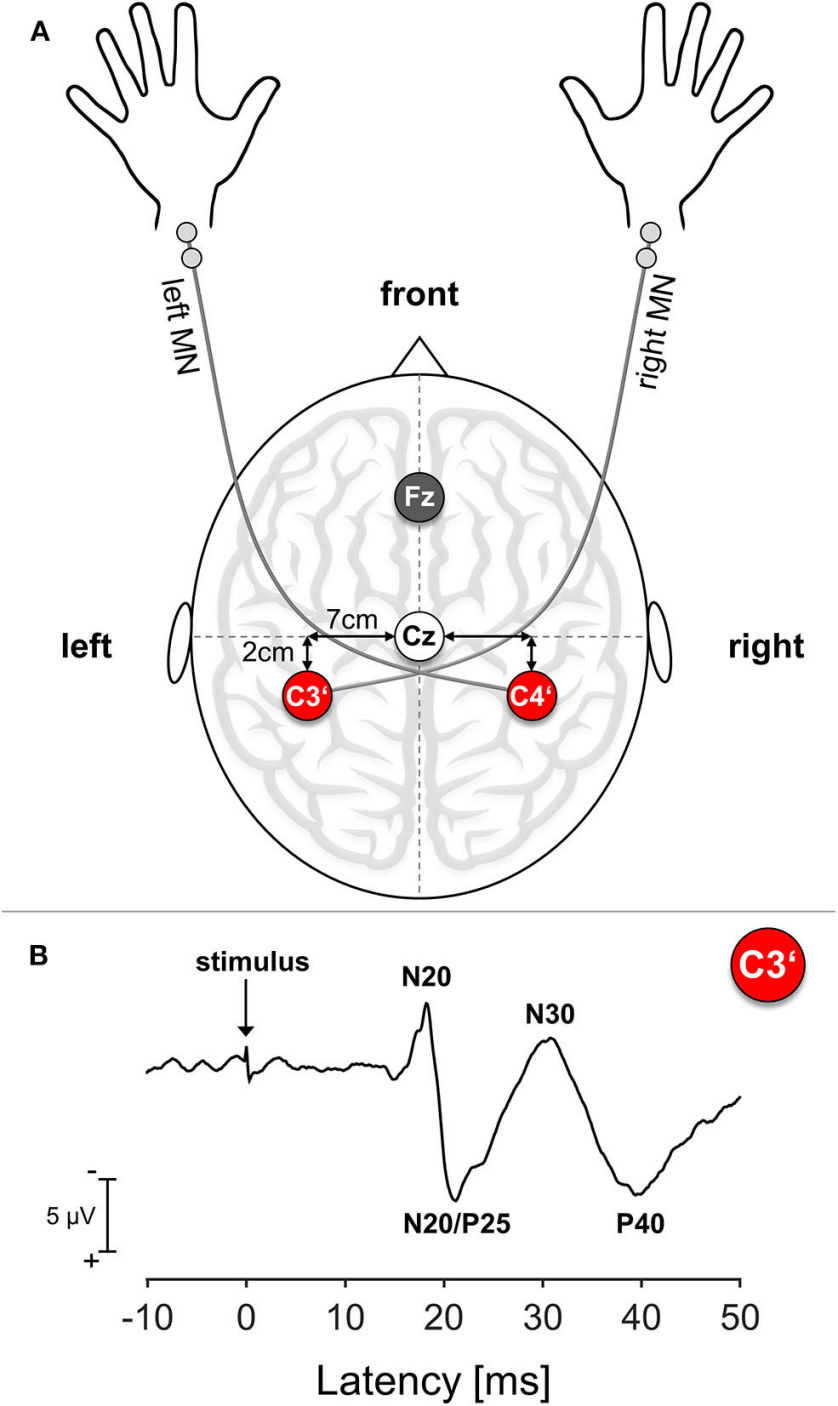
Somatosensory Evoked Potential (SEP): Setup and Exemplary Trace. **(A)** Schematic SEP electrode setup. MN, median nerve; Cz, Mid of the head; Fz, Reference electrode position; C3′, Electrode position corresponding to left S1; C4′, electrode position corresponding to right S1. **(B)** Exemplary SEP trace of a representative young participant. The arrow marks the time point of stimulus onset.

Cortical SEPs were recorded before and immediately after termination of the motor task. To relate efferent output with afferent input, we chose the right median nerve (MN) for electrical stimulation because of its innervation of the APB muscle. We confined ourselves to MN mono-stimulation because of the difficulties in obtaining suitable axillary nerve SEP responses as an afferent reference for the deltoid muscle. As a control condition and to relate to potential ipsilateral training effects, SEPs were also recorded over the untrained contralateral right S1, which relates to the left MN. Participants were seated in a darkened and quiet room. Standard block electrodes were placed on the right and left MN at the wrist. MN stimulation was performed using a pulse duration of 0.2 ms and a repetition rate of 3.1 Hz. Stimulation intensity was set to 2.5 times above the sensation threshold, which was determined before and after the training period. Subjects reported a non-painful prickling phenomenon in the thumb and index and middle finger of the stimulated hand to verify correct positioning of the stimulating electrode. SEPs were obtained from two scalp positions according to the 10–20 system, with silver disk electrodes attached with a conductive electrode paste. For obtaining impedance under 5 kΩ, the underlying skin area was cleaned with alcohol pads and roughened with exfoliating scrub. The hand presentation at S1 was marked 2 cm posterior to the C3 and C4 positions, corresponding to C3′ for stimulation of the right MN and C4′ for the left MN (Giblin, 1964). During recordings, the electrode Fz was used as a reference and an electrode at the right forearm served as a ground electrode; see also Figure 1A. In order to ensure recording from identical locations before and after the co-contraction task, EEG electrodes were not removed during learning. SEPs were acquired with a band-pass filter between 5 and 1,500 Hz and digitized with a sampling rate of 10,000 Hz (sampling interval 100 μs) in epochs from 20 ms before and 80 ms after stimulus onset. EEG recording was performed using an automated artifact rejection such that sweeps exceeding 100 μV were rejected from the analysis. A total of 300 valid stimulus-related epochs were registered and averaged for every trial.

### Data Analysis

Time differences between the onsets of the contractions of both muscles were calculated as markers for learning (MOA). Offline analysis of EMG data was performed using ProEMG (ProEMG 2.1.0.4, prophysics AG, Kloten, Switzerland). EMG signals were high pass filtered with a second-order Butterworth filter (12 dB/oct) with a cutoff frequency of 20 Hz. Teager–Kaiser energy operator (TKEO) for improved EMG onset detection was used (Solnik et al., 2010). For each signal, mean μ and standard deviation σ of the baseline were computed from 50 raw samples. For onset detection threshold T was determined as T = μ + *h*σ, where h is a preset factor, defining the level of the threshold (Solnik et al., 2010). After TKEO processing, T was set at *h* = 20 due to the very low magnitude of the baseline. The estimated onset time was identified as the first point when the rectified and smoothed signal (50 Hz low-pass filter) exceeded the threshold T for more than 20 ms. For subsequent statistical analyses, MOA were binned in 3 min intervals, resulting in a total number of 10 bins consisting of nine MOAs each.

For all subjects, the following SEP amplitudes with cortical origin were analyzed separately: N20, N20/P25 complex, N30, P40. The N20 amplitude was assessed as the difference between the onset and the first negative peak usually ranging around 17–22 ms after stimulus onset (Sonoo et al., 1996). The amplitude of the N20/P25 complex was measured as the difference between the N20 peak and maximum subsequent positivity. The N30 amplitude was measured as the difference between the N20/P25 complex peak and maximum subsequent negativity and the P40 amplitude as the difference between the N30 peak and maximum subsequent positivity; see also Figure 1B for details. Since the N60 component could not be reliably detected in all participants, we decided to not analyze this component.

### Statistical Analyses

Statistical analyses were conducted using the Statistical Software Package for Social Sciences (IBM SPSS Version 25). Demographic data were analyzed by comparing YAs and OAs regarding their LQ and the number of hours of sport per week and hours of sleep the night before the experiment. After testing the normality assumption using the Shapiro–Wilk test, independent-samples *t*-test or, in cases of non-normal distributions, Mann–Whitney *U*-test (MWU) was performed to compare the groups with regard to their demographics. Repeated-measures analyses of variance (RM-ANOVA) with factor GROUP (YA, OA) and TIME (pre–post training) were used to assess changes in VAS scores. We decided to normalize our behavioral data to the first bin performance to account for potential differences in initial performance between YAs and OAs. After checking our behavioral data for normality using the Shapiro–Wilk test, we performed an RM-ANOVA with the factors GROUP (YA, OA) and TIME (BIN1–BIN10). To additionally reveal effects of learning, we calculated each individual slope of learning from the non-normalized behavioral data and compared average slope values between groups using an independent-samples *t*-test. SEP amplitudes were analyzed separately for each component. We were primarily interested in the C3′ electrode components, since the motor task was performed with the right arm and we expected mostly contralateral left S1 contributions to the learning process. However, to account for unspecific effects of motor learning to the right S1, all C4′ components related to the non-trained left arm also were analyzed. Potential group differences were investigated using an RM-ANOVA with factor group (YA, OA) and factor time (Pre, Post) for each component separately for each hemisphere. To additionally investigate whether the initial size of the SEP amplitude correlates with the subsequent amount of learning, we performed a correlation analysis between each C3′ electrode component before training (SEP Pre) and the individual slope.

Partial eta-squares ($\eta_{p}^{2}$) are provided for relevant ANOVAs as measures of effect size. A *p*-value of < 0.05 was considered to be significant. Where necessary, data were Greenhouse–Geisser corrected and *p*-values were corrected for multiple comparisons.

## Results

### Demographics

Participants differed regarding their LQ, MWU: *U* = 323, *p* = 0.001, with YAs showing smaller LQ values compared with OAs (see also Table 1 for details). Participants also significantly differed regarding their total hours of sports per week, with YAs having more weekly sport hours than OAs, MWU: *U* = 114, *p* = 0.02. Participants did not differ with regard to their hours of sleep the night before the experiment, and all OAs met the criteria score of ≥27 in the MMSE. Both groups showed a significant reduction of attention over the course of the experiment, main effect TIME, *F*_1,38_ = 15.1, *p* < 0.001; however, the amount of change did not differ between groups, TIME^*^GROUP interaction, *F*_1,38_ = 0.31, *p* = 0.58. Fatigue also increased over the course of the experiment, main effect TIME, *F*_1,38_ = 12.55, *p* = 0.001, but with no differential effect across groups, TIME^*^GROUP interaction, *F*_1,38_ = 0.12, *p* = 0.73. Discomfort did not differ before and after the experiment, main effect TIME, *F*_1,38_ = 0.11, *p* = 0.75, in none of the groups (TIME^*^GROUP interaction, *F*_1,38_ = 0.11, *p* = 0.75; see Table 2 for details).

**Table 2:** Attention, fatigue, and discomfort assessed on a visual analog scale (VAS).

	**YA**		**OA**	
	**Pre**	**Post**	**Pre**	**Post**
Attention	8.20 ± 0.22	7.60 ± 0.23^*^	8.00 ± 0.21	7.55 ± 0.21^*^
Fatigue	7.70 ± 0.29	6.85 ± 0.28^*^	7.85 ± 0.26	7.15 ± 0.42^*^
Discomfort	1.15 ± 0.11	1.18 ± 0.11	1.05 ± 0.05	1.05 ± 0.05

*Attention scale scores ranged from 1 (no attention) to 10 (highest attention level), fatigue scale ranged from 1 (high fatigue level) to 10 (no fatigue), and discomfort scale scores ranged from 1 (no discomfort) to 10 (highest level of discomfort)*.

* *indicates significant differences (p < 0.05) in the variable time, independent of group belonging. All values are depicted as mean ± standard deviation of the mean. OA, older adults, YA, younger adults*.

### Behavioral Data: Motor Learning

**Figure 2:**
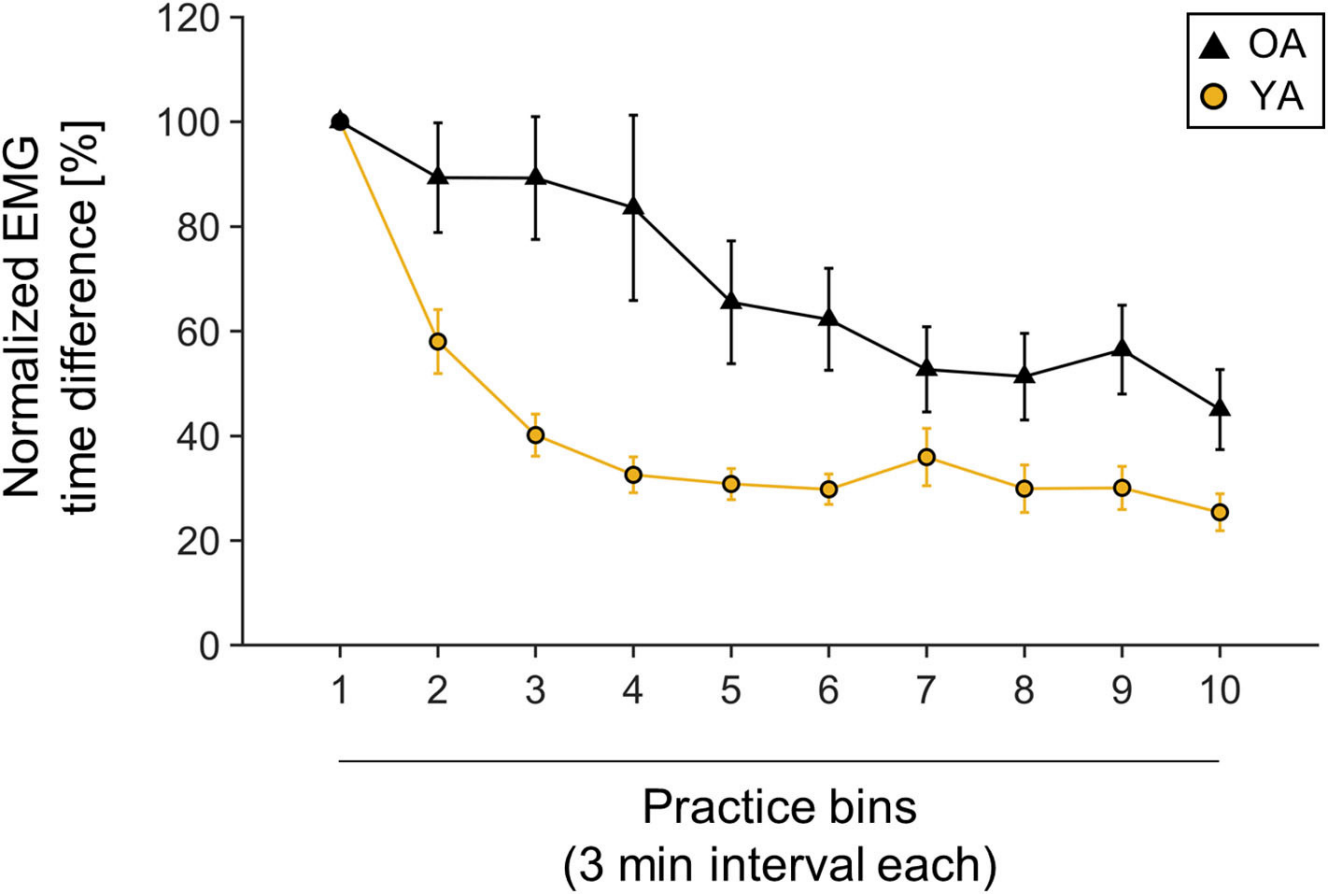
Muscle onset asynchrony (MOA) values normalized to first bin performance. One bin is calculated as one averaged 3-min interval. Yellow line indicates young participants' mean performance; black line indicates older participants mean performance. Mean values and corresponding standard deviation of the mean are depicted.

Behavioral data were normalized to first bin performance, since starting performance was very variable across participants, with YAs performing on average with 90.42 ± 60.9 ms and OAs with 69.41 ± 35.37 ms MOA [independent-samples *t*-test, *t*(30.51) = 1.33, *p* = 0.19]. Both groups significantly improved their performance over time, measured by shortened MOA [main effect TIME, *F*_(3.49,132.78)_ = 28.16, *p* < 0.001, $\eta_{p}^{2}$ = 0.43]. However, there was a differential effect of motor learning between YAs and OAs, with OAs showing higher MOA over the time course of learning [main effect GROUP: *F*_(1,38)_ = 10.92, *p* = 0.002, $\eta_{p}^{2}$ = 0.22]. Interestingly, the learning slope was not significantly different between YAs and OAs [independent-samples *t*-test, *t*(38) = −0.81, *p* = 0.42]. For visualization, see also Figure 2.

### Somatosensory Evoked Potentials

**Figure 3:**
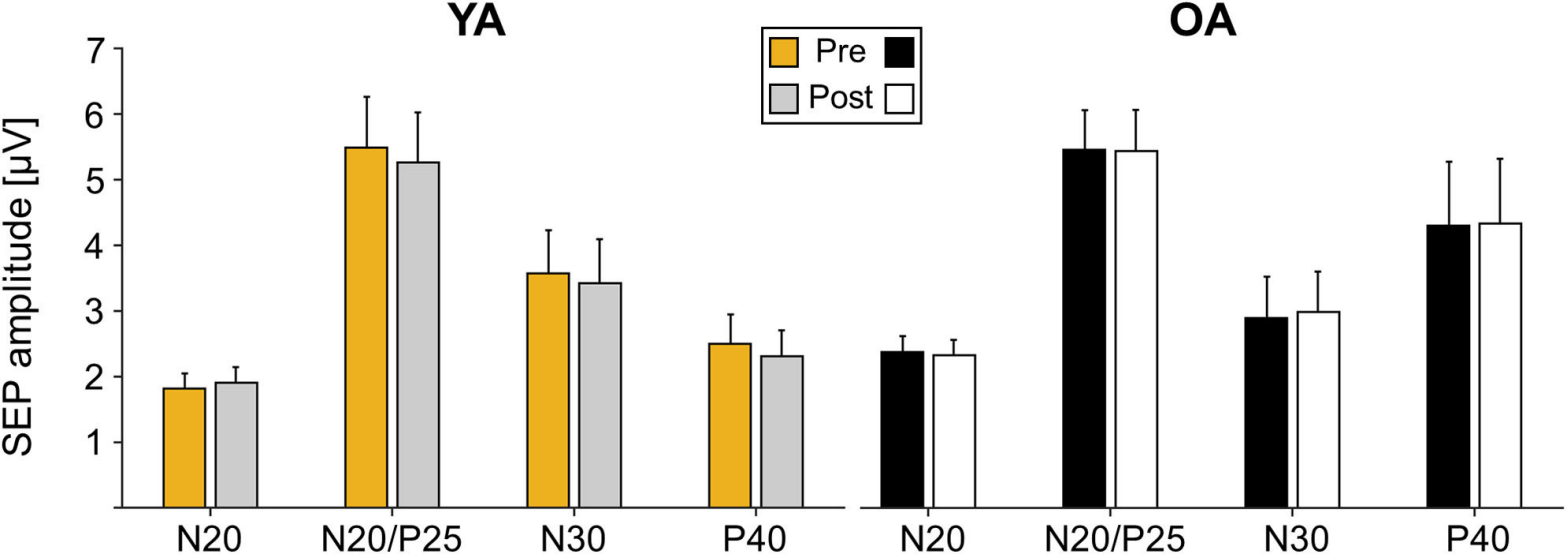
Somatosensory evoked potential (SEP) amplitudes pre and post motor learning in μV for electrode C3′ for young adults (YAs) and older adults (OAs). Yellow and gray bars represent YAs; black and white bars represent OAs. Mean SEP amplitudes and standard deviation of the mean are depicted.

Analyses of SEP components did not reveal significant age-related differences in the component N20, N20/P5, N30, and P40 prior to learning (all main effects GROUP *p* > 0.05). Also, none of the components significantly changed over time as a consequence of motor learning (all main effects TIME *p* > 0.05), and age also did not alter the amount of change over time (all TIME × GROUP interactions *p* > 0.05); see Table 3 for statistical details and Figure 3 for averaged SEP amplitudes.

**Table 3:** 2 × 2 Repeated-measures analysis of variance (RM-ANOVA) summary for all somatosensory evoked potential (SEP) components separated for C3′ and C4′ before and after motor learning.

**Component**	**Factor 1: group main effect**				**Factor 2: time main effect**			**Interaction: factor 1 × 2**		
	***F***	***P***	$\eta_{p}^{2}$	**(*df1, df2*)**	***F***	***p***	$\eta_{p}^{2}$	***F***	***p***	$\eta_{p}^{2}$
**C3′**										
N20	2.75	0.106	0.07	(1, 38)	0.06	0.814	0.001	0.67	0.417	0.02
N20/P25	0.01	0.963	0	(1, 38)	0.71	0.405	0.02	0.51	0.481	0.01
N30	0.57	0.454	0.02	(1, 38)	0.01	0.931	0.002	0.45	0.506	0.03
P40	2.81	0.102	0.07	(1, 38)	0.42	0.519	0.01	0.84	0.364	0.02
**C4′**										
N20	0.11	0.750	0.003	(1, 38)	0.22	0.650	0.01	1.35	0.251	0.03
N20/P25	0.001	0.977	0	(1, 38)	1.08	0.306	0.03	0.02	0.879	0
N30	0.14	0.710	0.004	(1, 38)	1.28	0.269	0.03	0.52	0.479	0.01
P40	3.07	0.089	0.08	(1, 38)	3.01	0.089	0.07	0.22	0.639	0.01

*Factor 1: YA, OA. Factor 2: Pre, Post. OA, older adults, YA, young adults*.

### Correlation Analyses

**Figure 4:**
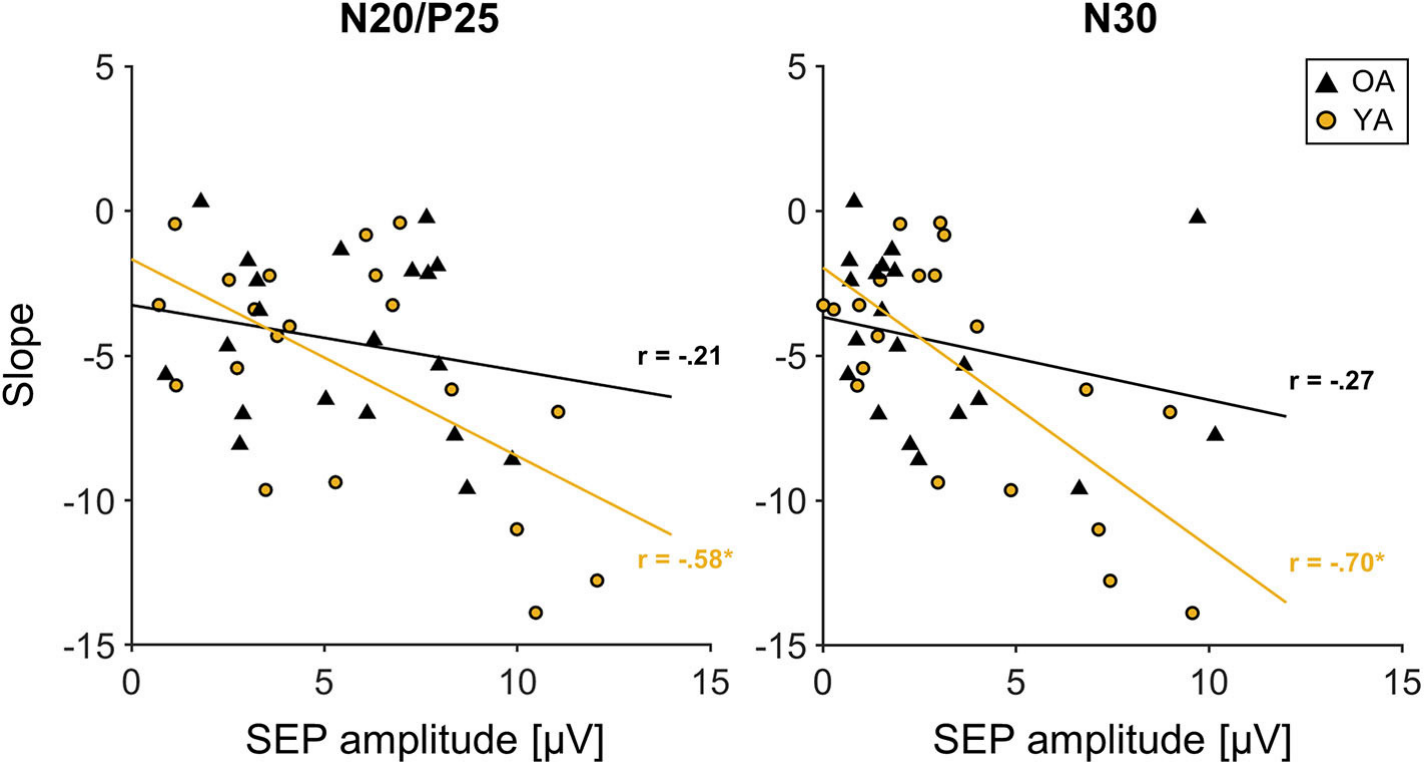
Correlation between initial somatosensory evoked potential (SEP) amplitude of the N20/P25 and N30 component derived from C3′ electrode (S1 contralateral to motor task performing hand) and slope. Young participants are depicted in yellow; older participants are depicted in black. Solid lines in corresponding colors represent linear trend lines for each age group. R: correlation coefficient for SEP amplitude and slope values depicted for each age group.

Correlation analyses across all participants between initial SEP amplitude and the time course of learning, measured as the individual slopes, revealed a significant correlation for the N20/P25 (*r* = −0.442, *p* = 0.004, *r*^2^ = 0.19) and the N30 component (*r* = −0.526, *p* < 0.000, *r*^2^ = 0.28). This correlation seems to be mainly driven by YAs, since groupwise analysis revealed a strong correlation for the N20/P25 (*r* = −0.58, *p* = 0.007, *r*^2^ = 0.34) and the N30 component (*r* = −0.7, *p* = 0.001, *r*^2^ = 0.49) only in YAs, while for OAs, correlations were much smaller (N20/P25: *r* = −0.21, *p* = 0.39, *r*^2^ = 0.04; N30: *r* = −0.27, *p* = 0.25, *r*^2^ = 0.07). More specifically, participants who showed the largest changes in MOA (a decrease in MOA indicates motor learning) were those with the largest baseline SEP amplitudes (N20/P25, N30). For visualization, see also Figure 4.

## Discussion

In the present study, our main objective was to examine whether motor learning differently activates the somatosensory system in YAs and OAs, measured by cortical SEP amplitude changes. We found that repetitive execution of a co-contraction task induced stable motor learning in YAs as well as in OAs. Interestingly, even though absolute MOAs in OAs were higher compared to those in YAs, the learning slope did not differ between age groups. This finding suggests that OAs showed intact learning capabilities even in complex motor learning scenarios as the co-contraction task. Furthermore, no changes were found in SEP amplitudes after motor learning in both groups. However, a correlation analysis revealed a positive association between baseline SEP amplitudes of the N20/P25 and N30 SEP component and the motor learning slope in YAs. Hence, the present findings suggest that SEP amplitudes might serve as a predictor for the individual motor learning success, at least in YAs.

Contrary to previous studies that combined the co-contraction task and SEP measurements (Schwenkreis et al., 2001; Pleger et al., 2003), we did not find any cortical SEP changes after execution of the motor task in both age groups. One reason for this divergent result may have been the shorter task duration of only 30 min compared with the abovementioned studies using a longer task duration of up to 1 h. We here used a shorter version of the co-contraction task because a significant learning effect was already seen after 30 min in previous studies (Schwenkreis et al., 2001; Pleger et al., 2003). However, based on our findings, performing the co-contraction task for only 30 min might have been insufficient to induce neuroplastic changes in S1 processing, since SEP amplitudes remained unchanged after motor learning. In a study by Andrew et al. (2015) in YAs that used motor tasks such as repetitive typing and tracing, an increase of SEP amplitudes was shown already after 10 min of training. However, when comparing the co-contraction task used in the present study with typing and tracing tasks from the study by Andrew et al. (2015), we consider the co-contraction task as more complex, since multiple joints are involved and the task requires a high precision regarding the timing of the movement. Therefore, we argue that in the co-contraction task, the somatosensory system is activated at a later time point in the motor learning process—potentially even later than 30 min of task performance. Another argument in this line is that different phases of motor learning are linked to distinguishable different recruited networks. Motor skills are typically learned slowly over multiple training sessions until performance reaches nearly asymptotic levels (Dayan and Cohen, 2011). Across different experimental paradigms, skill acquisition develops initially relatively fast and later more slowly, when further gains develop incrementally over multiple sessions of practice (Doyon and Ungerleider, 2002; Doyon and Benali, 2005). The relative duration of what can be defined as fast and slow learning is highly task specific. For example, the fast stage of learning a simple four-component key-press sequence could last minutes (e.g., Karni et al., 1995), whereas the fast stage of learning to play a complex musical piece may last months (for review, see Dayan and Cohen, 2011). Fast learning of sequential motor tasks decreases regional brain activity in the dorsolateral pre-frontal cortex, M1, and pre-supplementary motor area (Sakai et al., 1999; Floyer-Lea and Matthews, 2005). Pre-motor cortex, supplementary motor area, parietal regions, striatum, and the cerebellum show increased activation as learning progresses (Honda et al., 1998; Grafton et al., 2002; Floyer-Lea and Matthews, 2005). With long-term learning, increases in activity were found in the left S1 and M1 and in the right putamen (Floyer-Lea and Matthews, 2005). According to this, the process from early to late stages of motor skill learning is characterized by a relocation of activation from anterior to more posterior regions of the brain (Floyer-Lea and Matthews, 2005), which is thought to reflect a progressive decrease in reliance on attentional resources and executive function (Kelly and Garavan, 2005). Knowing this, a 30-min session of our co-contraction task potentially refers to the phase of fast motor skill learning, since no asymptotic level of performance was reached. To fully answer the question, whether a longer task duration may have resulted in a stronger S1 amplitude change, future studies are needed, which should also take into account that different motor learning stages exist, which are linked to different recruited networks.

Apart from these divergent SEP findings, we showed that the baseline SEP amplitudes could be used as a predictor for motor learning outcomes, at least in YAs. Larger initial SEP amplitudes were associated with stronger MOA reduction, indicating better muscle synchrony between trained muscle groups. The initial baseline SEP amplitude may serve as a measure of activation of the somatosensory system at the beginning of task performance. In this sense, higher activation in the somatosensory region is associated with higher motor learning success, a finding corresponding to a previous study, which found that higher baseline SEPs were associated with greater plasticity levels (Pellicciari et al., 2009). It is suggested that the N20 component is generated in the thalamo-cortical projection (Yamada et al., 1984) or at the posterior bank of the central sulcus, corresponding to Brodmann area 3b of S1. P25 and N20 originate from two different cortical sources. P25 is a radial dipole, located in Brodmann area 1 of S1, while N20 is a tangential dipole of S1 (Allison et al., 1991; Buchner et al., 1996). The source of the N30 component has been attributed to the motor cortex or the supplementary motor area (Waberski et al., 1999). Having a somatosensory-generated and a motor-generated signal predicting motor learning success corroborates the idea that S1 plays a key role in learning complex motor tasks and supports the idea of an interconnected sensorimotor system. The integration of the sensory and motor systems allows using sensory information for planning useful motor actions—what gives us flexibility to adapt to changing environmental conditions. However, it cannot be ruled out that higher slope values in our study originate from worse initial performance, since higher initial values potentially allow higher reduction rates. In this sense, higher baseline SEP amplitudes may also represent neural mechanisms compensating lower learning abilities rather than predicting learning success. Interestingly, no correlations were found in OAs. In line with previous arguments, one could speculate that areas other than S1 correspond to successful motor learning in the elderly, such as pre-frontal regions. There are studies suggesting that in old age, SEP amplitudes increase independent of task performance (Lüders, 1970; Kazis et al., 1983; Huttunen et al., 1999; Stephen et al., 2006). Contradictory to these findings, our results show no differences in SEP amplitudes between YAs and OAs neither at baseline level nor post motor learning. One reason for this divergent result could be that since a relatively heterogeneous older age group was tested, baseline variability in SEP amplitudes was too high to disentangle significant differences between age groups. Furthermore, due to our relatively strict inclusion criteria, our older study cohort could be healthier and more active compared with the average population of this age. However, fitness levels clearly showed differences between YAs and OAs, indicating nevertheless age-related differences. Also, the mean age of our OAs falls within the age range commonly tested (Lüders, 1970; Kazis et al., 1983; Huttunen et al., 1999; Stephen et al., 2006). Since also contradictory findings have been reported with regard to SEP amplitude in older age, suggesting smaller amplitudes in short-latency cortical potentials in OAs (Kakigi, 1987), no clear rationale can be provided about the effects of age on early SEP amplitudes.

### Study Limitations

In the present study, post-training SEP measurements were performed immediately after completing the co-contraction task. However, it might well be that motor learning induced aftereffects in the somatosensory system that develop just hours after active motor learning. These potential functional adaptations in S1 during the motor consolidation phase were not captured with the present study design. Therefore, we suggest that future studies should investigate motor learning-induced effects on SEP amplitudes not only directly after task completion but also at later time points to evaluate the potential progress of learning-induced neuroplasticity in S1. Additionally, we did not investigate the role of multiple motor learning sessions on cortical SEP amplitude changes and did not test for long-term effects. Our attempt was to investigate the effect of a short-term training intervention on S1 amplitude change, and therefore, we did not consider multiple testing. Thus, multiple repetitive co-contraction task sessions may have induced stronger behavioral effects that may have resulted in profound SEP changes. Furthermore, our aged study cohort was selected according to relatively strict inclusion criteria and can therefore be considered healthy and active. With a mean age of 67 years and no associated diseases, it may be questionable whether this cohort is a representative example of the population of OAs. Not only the duration of a motor task might affect changes in somatosensory processing, attention could have impacted too. In the present study, participants have shown enlarged fatigue and reduced attention after execution of the co-contraction task. Studies have shown that short-latency SEP components up to 50 ms post stimulus are not affected by attention (Papanicolaou et al., 1989; Arthurs et al., 2004; Ikeda et al., 2016). Therefore, we do not expect increasing levels of fatigue to have systematically influenced our SEP results. Earlier research has shown attenuation of SEP amplitude in higher-order somatosensory areas, such as secondary somatosensory areas (Frot et al., 1999), with increased attention (Papanicolaou et al., 1989; García-Larrea et al., 1995). However, because no late SEP amplitudes were tested in our study protocol, no conclusion can be made about the influence of attentional state on later SEP amplitudes. Furthermore, one additional limitation of the current study could be the exclusion of brain regions outside the somatosensory cortex, such as the posterior aspect of the parietal cortex and premotor cortex, which are obviously also involved in motor learning. However, our main interest was to investigate S1 activity related to motor learning in aging; therefore, no other brain region measurement was involved.

## Conclusions

Combining measures of motor learning with methods of neurophysiological examination, e.g., SEPs, in OAs seems to be important to advance the knowledge on how motor learning proceeds over the life span. Our results indicated that both YAs and OAs were able to learn a complex motor learning task and significantly increase synchrony after only 30 min of task performance. Furthermore, we were able to show that the baseline magnitude of the N20/P25 and the N30 SEP component predicted the amount of motor learning at least in YAs. We did not observe training-induced changes in SEP amplitudes neither in YAs nor in OAs, which is why future studies using longer-lasting multiple training sessions are needed. Shedding light onto this line of research will reveal further important insights into age-related changes in sensorimotor integration. This information might be of particular relevance for future studies that aim to maintain or prolong an independent lifestyle with advanced age in daily activities requiring learning new movement patterns. Not just active training, even passive sensory stimulation improves motor and sensory performance (Kalisch et al., 2008) in older ages. Furthermore, combining neurophysiological assessments of brain activation with behavioral outcome measures may help identify potential targets for supportive non-invasive brain stimulation approaches.

## Data Availability Statement

The data that support the findings of this study are available on request from the corresponding author. The data are not publicly available due to data protection policies practiced at our institute, e.g. their containing information that could compromise the privacy of research participants.

## Ethics Statement

The study was reviewed and approved by the local ethics committee of the medical faculty of the University of Leipzig, Käthe-Kollwitz-Straße 82, 04109 Leipzig. Participants provided written informed consent to participate in the study.

## Author Contributions

CP and PR designed the study. CP and MH collected the data. CP, DC, and EK analyzed the data. DC created the figures. CP and EK drafted the manuscript. DC, MH, AV, and PR provided critical revision. All authors approved the final version of the manuscript and agree to be accountable for all aspects of the work in ensuring that questions related to the accuracy or integrity of any part of the work are appropriately investigated and resolved. All persons designated as authors qualify for authorship, and all those who qualify for authorship are listed.

## Conflict of Interest

The authors declare that the research was conducted in the absence of any commercial or financial relationships that could be construed as a potential conflict of interest.

## Abbreviations

APB: abductor pollicis brevis
EMG: electromyography
GABA: ⋎-amino butyric acid
LQ: laterality quotient
MN: median nerve
MMSE: Mini Mental State Examination
MOA: muscle onset asynchrony
MWU: Mann–Whitney *U*-test
M1: primary motor cortex
OA: old adult
RM-ANOVA: repeated-measures analysis of variance
SEP: somatosensory evoked potential
S1: primary somatosensory cortex
TKEO: Teager–Kaiser energy operator
VAS: visual analog scale
YA: young adult.
